# Article 1: Supervision, Performance Assessment, and Recognition Strategy (SPARS) - a multipronged intervention strategy for strengthening medicines management in Uganda: method presentation and facility performance at baseline

**DOI:** 10.1186/s40545-016-0070-x

**Published:** 2016-05-31

**Authors:** Birna Trap, Denis Okidi Ladwar, Martin Olowo Oteba, Martha Embrey, Mohammed Khalid, Anita Katharina Wagner

**Affiliations:** Management Sciences for Health, Plot 15, Princess Anne Drive, P.O. Box 71419, Bugolobi, Kampala Uganda; Ministry of Health Uganda Pharmacy Division, Plot 6/Lourdel Rd, P.O. Box 7272 Kampala, Uganda; Management Sciences for Health, 4301 N. Fairfax Drive, Suite 400, Arlington, VA 22203 USA; Harvard Pilgrim Health Care Institute, 133 Brookline Avenue, 6th Floor, Boston, MA 02215 USA

**Keywords:** Medicines management, Supervision, Performance assessment, Pharmacy indicators, Medicines management indicators, Public sector, Uganda

## Abstract

**Background:**

Uganda introduced a multipronged intervention, the supervision, performance assessment, and recognition strategy (SPARS), to improve medicines management (MM) in public and not-for-profit health facilities. This paper, the first in a series, describes the SPARS intervention and reports on the MM situation in Uganda before SPARS (baseline).

**Methods:**

To build MM capacity at health facilities, health workers were trained as MM supervisors to visit health facilities, assess MM performance, and use the findings to provide support and standardize MM practices. Performance is assessed based on 25 MM indicators covering five domains: dispensing quality (7 indicators), prescribing quality (5), stock management (4), storage management (5) and ordering and reporting (4). From the end of 2010 to 2013, MM supervisors assessed baseline MM performance of 1384 government (85 %) and private not-for-profit facilities at all levels of care in about half of Uganda’s districts.

**Results:**

The overall MM baseline median score was 10.3 out of a maximum of 25 with inter-quartile range (IQR) of 8.7–11.7. Facility domain scores (out of a maximum of 5) were as follows: storage management, median score of 2.9 (IQR 2.3–3.4); stock management 2.3 (IQR 2.0–2.8), ordering and reporting 2.2 (IQR 1.3–2.5), and dispensing quality 2.1 (IQR 1.7–2.7). Performance in prescribing quality was 0.9 (IQR 0.4–1.4). Significant regional differences were found: overall scores were highest in the Northern region (10.7; IQR 9.2–12.4) and lowest in the Eastern region (9.6; (IQR 7.8–11.2) (*p* < 0.001). Overall scores did not differ by facility ownership; however, government facilities scored lower in dispensing and storage and higher in ordering and reporting. Hospitals scored higher overall and in domains other than prescribing and stock management. Districts classified a priori as having high capacity for implementing SPARS had higher scores at baseline compared to lower-capacity districts.

**Conclusion:**

Assessing and building national capacity in MM is needed in both private not-for-profit and government facilities at all levels of care. The indicator-based, multipronged SPARS assessment has been described here, while the strategy’s impact has yet to be documented.

**Electronic supplementary material:**

The online version of this article (doi:10.1186/s40545-016-0070-x) contains supplementary material, which is available to authorized users.

## Background

For a health care system to improve individual and population health, needed medicines must be available, accessible, affordable, and appropriately used [1]. To ensure these requirements, numerous pharmaceutical sector processes, including ordering products, managing stock and storage, and prescribing and dispensing medicines must be effective and efficient. These processes are complex and depend on many factors, such as the availability and wise use of money, human resources, and information, and management capacity [2, 3].

Despite Uganda’s long-standing commitment to ensuring universal access to essential medicines, the health system and the pharmaceutical supply chain continue to face many well-documented constraints [4]. For example, in 2009/2010, the availability of a basket of 22 vital items in public health facilities averaged 53 %, and the Ministry of Health reported that less than 10 % of all facilities had six vital indicator tracer medicines available [5, 6]. In 2013, only 35 % of public health care providers correctly diagnosed at least four of five common conditions,[7] and providers at only 1 % of health facilities provided the correct treatment for simple cough and cold [5]. Meanwhile, less than 8 % of 376 pharmacy posts in the public sector were filled, and 79 % of all facilities lacked shelves, making it impossible to manage medicines appropriately [5, 8, 9].

In general, access to medicines has been addressed through fragmented and vertical interventions without considering the broader health system [2]. In Uganda, a number of predominantly educational interventions have been implemented to strengthen the health care system and build capacity at district and facility levels [10–12]. However, these interventions have not produced significant or sustainable improvements in medicines management (MM) or access [5]. Combinations of educational, managerial, regulatory, and financial interventions and multimethod training approaches can improve health system practices [12–14]. Several studies have demonstrated that supervision and on-the-job training significantly increase health workers’ morale and performance in providing services and managing medicines [15, 16]). Supervision that is supportive is more effective than supervision that is punitive [17–19], and a strategy that combined rewards with performance assessment increased vaccine coverage and strengthened vaccine management at facility level [20, 21].

As a multidisciplinary group comprising members of government and non-governmental organizations that is implementing a strategy to improve the medicines situation in Uganda, we define MM as all the processes that support the implementation of the national medicines policy in ensuring that good quality essential medicines and health supplies (EMHS) are available and appropriately prescribed and dispensed at health facilities. Uganda’s Ministry of Health adopted the national supervision, performance assessment, and recognition intervention strategy (SPARS) to improve MM in government and private not-for-profit health (PNFP) facilities that combines several intervention approaches. Although similar elements have been recommended to improve performance of health workers [14, 22], a strategy that combines these five interventions to improve MM has not previously been described or implemented nationally.

### Uganda’s health system

Uganda had a population of 36.6 million people in 2014, with an average annual growth rate of 3.2 % per year; the estimated population will be about 44 million people in 2020 [23]. Communicable diseases such as HIV, malaria, lower respiratory infections, meningitis, and tuberculosis cause most years of life lost [24].

Both public and private for-profit providers deliver the country’s health care services. PNFP providers are considered part of the public sector. Under a decentralized health care delivery model, the Ministry of Health sets health policy and provides strategic direction, while local governments are responsible for service delivery. The National Drug Authority is the government arm responsible for assuring the quality of all medical products in the country through regulations of manufacturers, wholesalers, pharmacies, and drug shops.

In 2013, Uganda’s 112 districts had 5229 health facilities, of which 55 % were government-owned, 17 % were PNFP, and 28 % private for-profit [23, 24]. Uganda’s public sector consisted of two government national referral hospitals, 14 regional referral hospitals, 144 general hospitals, 197 health centers (HC) level 4, 1289 HC3 and 2941 HC2 facilities, and more than 25,000 village health teams regarded as HC1 [25]. The government-owned National Medical Stores (NMS) supplies EMHS to all government health facilities. NMS uses a combination of a “pull” ordering system for hospitals and HC4 facilities and a “push” system, whereby central-level decision makers determine the types and quantities of medicines that HC3 and HC2 facilities will receive in a kit, with amounts depending on facility level. The Joint Medical Stores is a private not-for-profit medical supplier owned by the medical bureaus (Catholic, Protestant, Muslim, and Orthodox), which provides medicines for its PNFP facility customers using a pull-based distribution system for facilities at all levels of care. Both warehouses distribute EMHS directly to health care facilities.

Per capita expenditure on EMHS in 2013/14 was US$2.40, of which US$0.99 was for basic EMHS (up from US$0.50 in 2010/11), and the remaining US$1.41 was spent mainly on HIV, tuberculosis, and malaria commodities. Funding of EMHS is heavily dependent on donor funds, which covered 77 % of EMHS costs in 2013/14 [8].

The objectives of this paper, the first in a series of papers on SPARS, are to a) describe the components of SPARS, an innovative multipronged intervention strategy to improve MM in Uganda, and b) report on the MM situation in Uganda before the introduction of SPARS (other than its assessment tool). This paper thus describes the baseline MM situation in Uganda prior to SPARS implementation. Additional papers will describe the SPARS intervention feasibility and impacts on MM at health facilities over time.

## Method

This section describes SPARS and its components followed by detailing the district and facility selection and collection of baseline facility performance data using the SPARS tool.

### Supervision, performance assessment, and recognition strategy

SPARS is based on the theory that combining different interventions increases the likelihood of positive change. The strategy, which was nationalized in 2012, includes educational, managerial, regulatory, and financial interventions combined with performance assessment. MM supervisors (MMS) provide on-the-job supervision and mentoring of health workers. They also give managerial support to staff in the form of manuals and tools needed to standardize MM practices. Performance assessment focuses on 25 MM indicators measured at baseline and at each following supervisory visit to guide support and ensure evidence-based decision making. On the regulatory side, SPARS helps facilities pass the National Drug Authority’s inspections to license health facility pharmacies. Recognition in the form of reward items for health facilities, district health officers, and the MMS are part of SPARS.

#### Supervision

MMS who implement SPARS are district-level health care staff members who are employed by the government. District health officers select MMS based on their leadership and management skills and interest in and knowledge of pharmaceutical issues. Each district has one district MMS and two to five sub-district MMS who could be clinical officers, nurses, midwives, pharmacy staff, or storekeepers. In addition to their other duties, the district MMS oversee the sub-district MMS and also supervise the district hospital and HC4 facilities, while sub-district MMS supervise public sector HC2 and HC3 facilities. District health officers monitor performance of MMS with oversight from regional pharmacists and the Ministry of Health’s Pharmacy Division.

MMS receive 2 weeks of training and pass an exam at Makerere University in managing medicines, problem-solving, communication, and how to mentor health care workers and assess performance using the indicator-based tool. MMS who passed the exam receive 1 week of practical training in the field. The MMS are provided a netbook to enter the findings from the performance assessment and they receive 3 days of training in the use of the netbook and the electronic performance assessment tool. To increase their computer skills, we provide flash drives with self-paced learning aids about various software packages and other technologies.^1^ To facilitate MMS’ travel to their facilities, which are often in rural areas with rutted dirt roads, they receive motorbikes, riding gear, training, and examination in defensive riding. Once MMS pass their defensive riding exam, they are ready to provide regularly scheduled on-the-job training and supportive supervision visits at their assigned facilities.

District MMS and health sub-district MMS are expected to complete three and five supervisory visits per month, respectively. To standardize the time between visits, a facility should receive a visit every other month. After five visits, the interim time can be increased to every 4 months to maintain acceptable performance.

In addition, managerial tools to facilitate the supervision are provided to the MMS and to the supervised facilities. An EMHS management manual that describes procedures for receiving and storing medicines and supplies, completing order discrepancy reports, filling out stock cards, completing the stock book, conducting stock counts, and dispensing medicines is distributed to all health facilities and MMS. Other tools include stock cards, stock books, dispensing logs, and standard operating procedures. MMS also receive laminated job aids to guide their explanations of how to correctly dispense medicines and use the dispensing guidelines. A supervisory book is placed at the facility and filled out by the MMS at each visit, recording findings and agreed next steps. A white board in the pharmacy displays a spider graph with results from the performance assessment and progress between visits in the five MM domains. To motivate, coordinate, and strengthen SPARS implementation, MMS and district health officers attend biannual regional meetings and district meetings where they discuss national and district SPARS performance reports.

In 2013, the National Drug Authority introduced regular inspections of government and PNFP facility pharmacies to assess their adherence to good pharmacy practices. SPARS has been shown to help facilities prepare for these inspections, and there is 73 % overlap of the indicators used in the good pharmacy practices inspection tool and the SPARS performance assessment tool [26].

#### Performance assessment

**Fig. 1:**
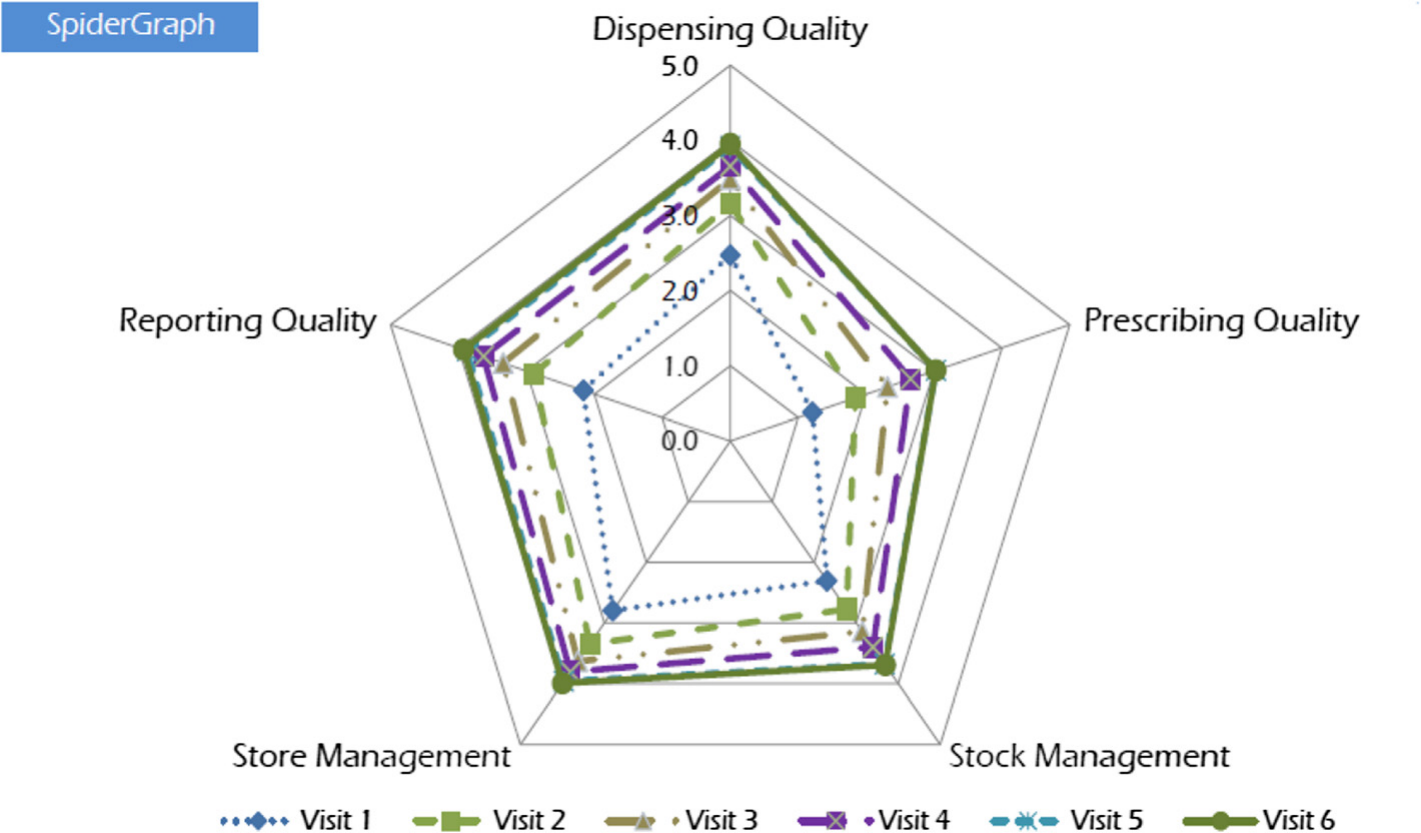
Spider graph of facility performance scores. Each facility has a spider graph printed on a white board that can be displayed in the pharmacy. The graph depicts performance progress between visits in the five MM assessment domains and functions as a management tool. This spider graph depicts facility scores for visits 1 to 6

Using evaluation as a management tool is well known [27]. MMS use record reviews, observation of staff practices, and patient exit interviews to assess and evaluate performance based on 25 MM indicators. The MMS note assessment results in the supervisory book and in the spider graph (Fig. 1). While in the field, they manually fill out a data collection form (Additional file 1) or (starting early 2012) use an identical electronic form on a netbook and submit it when they are able to access the Internet. Because many of the MMS were not computer literate at the start of the program, we designed and piloted an electronic form in the same format as the paper form and provided targeted computer training.

The SPARS performance assessment uses practical performance indicators to flag areas for improvement in a real-life setting, guide and focus the supervision and provide the health staff with an understanding of their facility’s issues and achievements. The 25 indicators are classified into five MM domains 1) dispensing quality, 2) prescribing quality, 3) stock management, 4) storage management, and 5) ordering and reporting.

The indicators chosen for the SPARS tool were based on tools used globally to assess pharmaceutical sectors, MM problems identified previously in surveys of the pharmaceutical sector in Uganda, and on an understanding of the processes needed in a system for ensuring that EMHS are available, of good quality, and used appropriately, in line with Uganda’s essential medicines policy. The rational drug use and the dispensing or patient care indicators are similar to validated World Health Organization (WHO) drug use core indicators [28]. In addition, complementary WHO indicators to measure adherence to standard treatment guidelines and the stock and storage management indicators have been validated and used globally [16, 28, 29]. To get an indication of how well staff is adhering to standard treatment guidelines in the time available for the assessment visit, and as a majority of facilities have only a few prescribers, we reduced the number of records required for review to 10 instead of the 30 to 100 recommended by World Health Organization [28].

To facilitate using the assessment as a management tool, each of the five domains has a maximum score of 5; therefore, the overall SPARS score has maximum score of 25. However, the number of indicators per domain varies from four to seven, so the contribution of an individual indicator to a domain score of 5 varies; for example, if the domain has five indicators, each is worth one point; if the domain has seven indicators, each is worth 5/7th of a point. If an indicator is not assessed for a facility, that indicator score is not included in the domain score calculation (rather than given a score of “0”). For example, if a facility did not have a score for one of the seven dispensing quality indicators (marked “not applicable”), then each of the six remaining indicators is worth 5/6th of a point instead of 5/7th of a point. MMS create a spider graph with a facility’s domain scores (Fig. 1) as a visual representation of a facility’s performance at each visit, which is useful for supportive supervision and performance tracking of the facility.

Additional file 1 in the supplementary file includes the data collection tool that was used in both manual and electronic versions. The tool describes each indicator and its scoring. Additional file 2 in the supplementary file describes the indicators by domains. Scores for indicators are composites of scores of sub-indicators and aggregated to scores that range from 0 to 1. Indicator, domain, and overall scores are primarily for comparing scores within facilities across visits.

To manage the country SPARS data, we developed a centralized data hub, called the pharmaceutical information portal, for storing, analyzing, disseminating, and reporting SPARS data. The hub aggregates the data that MMS submit, so that users can generate and share national and district reports.

Each of the 1384 facilities in the sample has an overall SPARS score and five domain scores. However, because not all facilities assessed every indicator at baseline, the number of facilities contributing to each indicator score varies from 33 to 1384.

#### Recognition scheme

The SPARS recognition component is a way to motivate the district health officers, MMS, and health facility workers and acknowledge progress in managing medicines. The rewards and linkage to performance was decided in discussion with health system officials at the start of SPARS based on identified needs and what was doable within rules and regulations. Most rewards are only given once; some are given annually (i.e., mobile telephone airtime and payment for each submitted SPARS performance assessment report). Rewards are largely linked to performance; for example, MMS who pass the training course receive a bag with pens, a calculator, and a netbook; when they pass the driving license and defensive riding tests, they receive the riding gear, motorbike, and motorbike license; after a specified number of supervisory visits, MMS are recognized with telephone time, etc. Other recognition items for MMS and district health officers include Internet airtime and payment for expenses linked to the SPARS visits they make. Over time, we simplified the expense payments per visit; now MMS receive UGX 30,000 (US$12) when they submit a SPARS visit report to cover fuel, food, and minor motorbike repair. We also provide funds annually for major motorbike repairs, servicing, and new tires.

Similarly, facilities that achieve a certain score—for example, 3 out of 5 in dispensing quality—receive a measuring cylinder, plastic dispensing bottles, and stainless steel tumblers for drinking water. When expiry records are available and updated and expired medicines are stored separately, the facilities are acknowledged with five mugs and 10 pens. Other things that help them deliver quality pharmacy services include tablet counting trays, copies of the *Uganda Clinical Guidelines*, soap, wall clocks to help track dispensing time, wall thermometers, masking tape to mark shelves, permanent markers, cleaning supplies, rat traps, pens, rulers, and ring binders. We also provide items for personal use such as T-shirts, calendars, toilet paper, sugar, tea, and mugs. The implementation of the SPARS was made possible through donor support by the United States Agency for International Development (USAID).

### Selection of districts and facilities

In 2009, we approached district health officers from the then 80 districts in Uganda regarding their interest in implementing SPARS. The overall response rate was 81 % (*n* = 65/80) and lowest in the Northern region with 76 %, followed by Central (81 %), Western and Eastern (84 %) regions. We ranked responsive districts according to their commitment to improving the availability of EMHS and scored their estimated capacity to carry out SPARS based on six evaluation criteria: district profile (size, population, number of facilities, Internet connectivity); infrastructure (district store size and condition); EMHS (availability and district distribution issues and solutions); partners (number and type of other development partners in the district); management and finance (per capita EMHS budget and expenditures); and staff (number of pharmaceutical staff members). Based on their scores, we classified their estimated capacity into “high,” “medium,” and “low” strata.

We then randomly selected 44 districts from the three strata (high, medium, and low) using systematic sampling of 20, 12, and 12 districts, respectively, and checked that all four regions were fairly equally represented; one more Western district was later selected randomly from all districts to reach a total of 45 districts, resulting in 15, 13, 9, and 8 districts from the Western, Eastern, Northern, and Central regions, respectively.

Government and PNFP facilities within districts were selected for inclusion by the MMS. The district-MMS selected the higher-level facilities (hospitals and HC4), and the sub-district MMS selected HC3 and HC2 for SPARS visits. The guiding principle was for an MMS to select five facilities to visit during the first month and another five during the following month. In the third month, the MMS would revisit the initial five facilities, and in the fourth month, revisit the second five facilities, and so on. In principle and with time, all facilities are SPARS supported, but the time it takes before all facilities receive a first visit depends on both the number of visits the MMS can make every month and the number of facilities under his or her responsibility, which ranges from four to more than 20. The intent was that during the first year, each MMS would cover at least 10 facilities, and visit the rest in the following year(s).

We also randomly selected another nine districts from the remaining 21 districts that had responded to the expression of interest. Sampling included two districts from three regions and three districts from the Central region. The selected districts represented high, medium, and low strata (1, 4, and 4, respectively). The nine districts would not be exposed to SPARS, so they could later serve as comparison facilities for assessing the impact of the SPARS intervention. In each of the nine districts, we included the district hospital and randomly selected one HC4 (when possible), three HC3, and two HC2 facilities for a total of 63 government and PNFP facilities, although ultimately, we only included 61 facilities due to incomplete data collection. In total, 15 % of intervention and 9 % of control facilities were PNFP. As a national strategy, SPARS will eventually be rolled out to include all districts in Uganda.

### Statistical analysis

We calculated measures of central tendency (medians, means) together with inter-quartile ranges (IQR) for the five domains and for the 25 indicator scores across categories of background characteristics. We used nonparametric equality of medians testing using Pearson’s chi-square test to determine whether median scores differed significantly across categories, because the domain scores were not normally distributed (based on results of the Shapiro-Wilk test). We present means and medians to illustrate skewness of the data. We used STATA software version 13 to perform all statistical analyses.

### Ethical considerations

This study describes a national capacity-building strategy and reports medicines management data collected by MMS under the Ministry of Health. The study did not involve human subjects or use personal data. As it constituted a Ministry of Health-initiated system intervention, no ethical review was required.

## Results

### Facilities with baseline assessments

From the last months of 2010 to 2013, 1499 health facilities had an initial SPARS visit to determine baseline MM scores. Because only 17 facilities received visits in 2010, we combined 2010 and 2011 data. Only 1384 (92 %) facilities were included in the baseline analyses due to lost reports or incomplete scores. A score was *not applicable* if, for example, the facility did not yet have the stock book; it was marked *missing values* if the store room was locked making the data inaccessible. Only three facilities had baseline values for all 25 indicators, and 83 % had values for at least 21 indicators. More than 5 % of the facilities had no values for seven indicators. The completeness of indicator recording improved somewhat over the course of baseline assessments and with the addition of electronic data collection.

We report baseline scores in overall medicines management and each of the five domains from 1384 facilities. More than half of the supervised facilities were government owned (85 %) and HC2 level (58 %). Table 1 shows that across the four regions proportions of facilities were comparable with regard to ownership and level of care but were not comparable with respect to year of baseline assessment and district capacity rank.

**Table 1:** Characteristics of health facilities at the baseline assessment, by region

	Western		Eastern		Central		Northern		Total		Chi-square *p*-value
	No	%	No	%	No	%	No	%	No	%	
Total	550	100	383	100	273	100	178	100	1384	100	
Ownership											
GOVT	472	85.8	319	83.3	226	82.8	158	88.8	1175	84.9	0.244
PNFP	78	14.2	64	16.7	47	17.2	20	11.2	209	15.1	
Level of care											
HC2	319	58.0	227	59.3	155	56.8	103	57.9	804	58.1	0.159
HC3	171	31.1	118	30.8	93	34.1	61	34.3	443	32.0	
HC4	47	8.5	19	5.0	18	6.6	7	3.9	91	6.6	
Hospital	13	2.4	19	5.0	7	2.6	7	3.9	46	3.3	
Year											
2011	214	38.9	300	78.3	244	89.4	65	36.5	823	59.5	<0.001
2012	235	42.7	80	20.9	26	9.5	105	59.0	446	32.2	
2013	101	18.4	3	0.8	3	1.1	8	4.5	115	8.3	
District Rank											
Good	353	64.4	196	54.4	144	53.1	133	75.1	826	60.9	<0.001
Average	26	4.7	116	32.2	71	26.2	0	0.0	213	15.7	
Poor	169	30.8	48	13.3	56	20.7	44	24.9	317	23.4	

*GOVT* government, *PNFP* private not for profit, *HC* health center; year indicates the year of the baseline assessment visit, district rank indicates the level of capacity of a district to implement the SPARS interventions

### Overall and five domain scores of MM

**Fig. 2:**
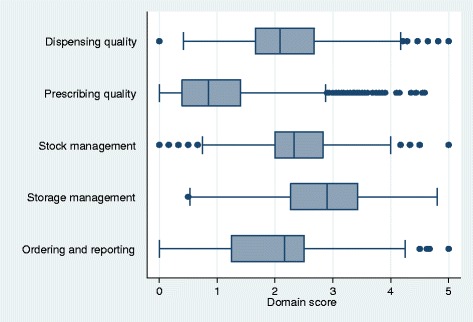
Box and whisker diagram of baseline domain performance scores of 1384 public health facilities in Uganda, 2010–2013. The figure displays the distribution of scores in the five domains. Shown are the minimum scores excluding outliers (first whisker - 25^th^ percentile -1.5*interquartile range [IQR]) and maximum score excluding outliers (last whisker –75^th^ percentile +1.5*IQR); the first quartile (lower part of the box), the median (line in box), and the third quartile (upper part of the box) and dots show the outlying scores. The spaces between the different parts of the box indicate the degree of dispersion (spread) and direction of skewness in the data for each of the five domains (on a scale of 0–5)

Generally, medicines management in Ugandan facilities was weak. The median overall performance score at baseline was 10.3 out of 25 (41 %). However, a few facilities scored close to 20 and one facility had a score close to 25, which is the maximum score possible. Scores for the five domains are given in Fig. 2 and Table 2. Facilities had high median scores (of 5) in the domain of storage management (median 2.9, IQR 2.3–3.4) and stock management (2.3, 2.0–2.8). Scoring in prescribing quality was poor (0.9, 0.4–1.4).

**Table 2:** Baseline performance scores, overall and in 5 medicines management domains, by public sector facility characteristics, Uganda, 2010–2013

Characteristics	Facilities %	Overall		Dispensing quality		Prescribing quality		Stock management		Storage management		Ordering and reporting	
		Median (IQR); Mean^a^	Chi-square test^b^	Median (IQR); Mean	Chi-square test	Median (IQR); Mean	Chi-square test	Median (IQR); Mean	Chi-square test	Median (IQR); Mean	Chi-square test	Median (IQR); Mean	Chi-square test
Region													
Western	40	10.5 (9.0–11.9); 10.6	<0.001	2.0 (1.6–2.5); 2.1	<0.001	1.1 (0.6–1.7); 1.3	<0.001	2.3 (2.0–2.8); 2.4	<0.001	3.0 (2.4–3.6); 2.9	<0.001	2.2 (1.3–2.5); 2.0	0.369
Eastern	28	9.6 (7.8–11.2); 9.6		2.1 (1.6–2.7); 2.2		0.5 (0.4–1.1); 0.8		2.3 (1.8–2.7); 2.3		2.5 (1.8–3.1); 2.5		2.2 (1.3–2.5); 1.9	
Central	20	10.6 (9.2–11.8); 10.6		2.3 (1.8–2.7); 2.3		0.5 (0.3–1.0); 0.7		2.5 (2.2–3.0); 2.5		3.0 (2.6–3.5); 3.0		2.2 (1.3–2.5); 2.1	
Northern	13	10.7 (9.2–12.4); 10.8		2.2 (1.8–2.9); 2.3		1.0 (0.6–1.4); 1.1		2.3 (1.9–2.8); 2.3		3.0 (2.5–3.4); 3.0		2.2 (1.3–2.5); 2.0	
Ownership													
Govt	85	10.2 (8.7–11.7); 10.3	0.084	2.1 (1.6–2.5); 2.1	<0.001	0.9 (0.4–1.4); 1.0	0.839	2.3 (2.0–2.8); 2.4	0.536	2.8 (2.2–3.4); 2.8	<0.001	2.2 (1.3–2.5); 2.0	<0.001
PNFP	15	10.6 (8.9–12.3); 10.7		2.7 (2.0–3.1); 2.6		0.8 (0.4–1.4); 1.0		2.3 (1.8–2.8); 2.2		3.3 (2.7–3.8); 3.2		1.7 (1.3–2.5); 1.7	
Level of Care													
HC2	58	10.2 (8.6–11.7); 10.3	<0.001	2.1 (1.7–2.7); 2.2	0.004	0.9 (0.4–1.6); 1.1	0.124	2.3 (2.0–2.8); 2.4	0.135	2.8 (2.1–3.3); 2.7	<0.001	2.2 (1.2–2.5); 1.9	0.272
HC3	32	10.1 (8.7–11.5); 10.2		2.1 (1.6–2.5); 2.1		0.7 (0.4–1.2); 0.9		2.3 (1.8–2.8); 2.3		2.9 (2.3–3.5); 2.9		2.2 (1.2–2.5); 2.0	
HC4	7	11.0 (9.7–12.4); 11.0		2.0 (1.5–2.8); 2.1		0.7 (0.4–1.1); 0.9		2.5 (2.2–3.0); 2.5		3.3 (2.7–3.7); 3.3		2.3 (1.5–3.0); 2.2	
Hospital	3	11.6 (11.0–13.1); 11.9		2.7 (2.1–3.3); 2.8		0.7 (0.4–1.1); 0.9		2.5 (1.8–3.0); 2.5		3.7 (3.1–4.2); 3.6		2.5 (1.3–2.8); 2.2	
Baseline Assessment Year													
2010/11	60	10.1 (8.6–11.5); 10.1	0.002	2.1 (1.6–2.7); 2.1	0.860	0.7 (0.4–1.1); 0.8	<0.001	2.3 (2.0–2.8); 2.4	0.699	2.8 (2.2–3.4); 2.8	0.553	2.2 (1.3–2.5); 2.0	0.019
2012	32	10.4 (8.8–12.0); 10.6		2.1 (1.7–2.7); 2.2		1.0 (0.6–1.7); 1.2		2.3 (2.0–2.8); 2.4		3.0 (2.3–3.5); 2.9		1.8 (1.3–2.5); 1.9	
2013	8	11.0 (9.9–13.4); 11.5		2.1 (1.7–2.9); 2.3		1.7 (1.2–2.4); 1.8		2.3 (1.8–2.8); 2.3		2.9 (2.2–3.6); 2.9		2.5 (1.3–2.5); 2.2	
Estimated District Capacity													
High	61	10.4 (8.9–11.9); 10.6	0.009	2.1 (1.7–2.7); 2.2	0.089	0.9 (0.5–1.4); 0.8	<0.001	2.3 (2.0–2.8); 2.4	0.474	2.9 (2.3–3.5); 2.9	0.014	2.2 (1.3–2.5); 2.1	0.010
Medium	61	9.6 (7.7–11.4); 9.7		2.1 (1.5–2.7); 2.2		0.5 (0.3–1.1); 1.1		2.3 (1.8–2.8); 2.3		2.7 (2.0–3.3); 2.7		2.0 (1.3–2.5); 1.8	
Low	23	10.4 (8.8–11.4); 10.2		2.0 (1.6–2.5); 2.1		0.9 (0.4–1.5); 1.1		2.3 (2.0–2.8); 2.3		3.0 (2.3–3.5); 2.9		1.8 (1.3–2.5); 1.9	
Total	100	10.3 (8.7–11.7); 10.4		2.1 (1.7–2.7); 2.2		0.9 (0.4–1.4); 1.0		2.3 (2.0–2.8); 2.4		2.9 (2.3–3.4); 2.8		2.2 (1.3–2.5); 2.0	

The maximum overall score is 25; the maximum score on each domain is 5

*IQR* interquartile ranges, *PNFP* Private not-for-profit facilities, *Govt* Government facilities

^a^medians and means are presented to allow for easy assessment of distributions; analyses are only conducted on medians

^b^testing equality of medians

As shown in Table 2, the median overall scores were around 10/25 and varied significantly by region (*p* < 0.001). Similar medians of overall scores were found in government-owned and PNFP facilities (10.2 vs. 10.6, NS). Median overall scores depended on level of care (HC3 facilities: 10.1; HC2: 10.2; HC4: 11.0; and hospitals 11.6 [*p* < 0.001]). Over the 4-year period during which the baseline visits occurred, the median overall SPARS scores differed (10.1 in 2010/11; 10.4 in 2012; 11.0 in 2013 [*p* = 0.002]). Median SPARS scores of facilities in districts based on pre-study capacity also differed (10.4 among those with high capacity, 9.6 among those with medium capacity, and 10.4 among those with low capacity), corresponding to MM practices across facilities in districts with a different capacity levels (*p* = 0.009).

#### Dispensing quality

Seven indicators (each with a possible maximum score of 1), are used to assess dispensing quality (Table 3). Dispensing time of less than 30 s is scored 0, between 31 and 60 s is scored 0.5, and 61 s or above scored at the maximum score of 1 (Additional file 1). The median score was 0.0 (IQR 0.0–0.5), indicating that dispensing time was too short to ensure good practices. About three-quarters of the facilities had a score of 0.5 that measured the availability of appropriate *packaging materials,* such as dispensing envelopes and containers, but only a quarter scored 0.5 for availability of *dispensing equipment,* such as a counting tray, spatula or spoon, and measuring cylinder. About a quarter of the facilities scored 0.8 or more on availability of *dispensing services* including, chairs, privacy, hand-washing, and drinking water. About three-quarters of the facilities had a score of 0.5 on *patient care*, which is a measure of any discrepancy between prescribed and dispensed medicines and the patient’s knowledge of how much medicine to take, how often, how long, and the reasons for taking the medicine. *Labeling* assesses whether medicines were labeled with the medicine’s name, strength, quantity, date, dose, patient name, and facility name, for which a quarter of the facilities had a score of 0.3. *Rationing of antibiotics* occurred if the patient received less than a full course of amoxicillin or co-trimoxazole. Rationing occurs when a facility is running out of stock or if a patient cannot pay for a full course, and the indicator’s median score was 1 as rationing rarely occurs. Median dispensing scores differed significantly by regions, facility types, and levels of care. Dispensing quality median scores did not differ across years of baseline assessments or estimated levels of district capacity (Table 2).

**Table 3:** Baseline performance scores on 25 medicines management indicators of public sector facilities in Uganda, 2010–2013. Maximum and best score for each indicator is 1

Indicator	Number of facilities	Median (IQR); Mean
Dispensing quality		
1. Dispensing time	1356	0.0 (0.0-0.5); 0.3
2. Packaging material	1383	0.5 (0.5-0.5); 0.5
3. Dispensing equipment	1381	0.0 (0.0-0.5); 0.3
4. Services available at dispensing domain	1384	0.5 (0.5-0.8); 0.6
5. Patient care	1341	0.5 (0.5-0.8); 0.6
6. Labeling	1344	0.0 (0.0-0.3); 0.1
7. Rationing of antibiotics	803	1.0 (1.0-1.0); 1.0
Prescribing quality		
8. Correct use of prescription recording system	1333	0.0 (0.0-0.5); 0.2
9. Rational prescribing	1380	0.4 (0.2-0.4); 0.4
10. Adherence to standard treatment guidelines for diarrhea	1014	0.0 (0.0-0.0); 0.1
11. Adherence to standard treatment guidelines for cough and cold	1272	0.0 (0.0-0.0); 0.1
12. Adherence to standard treatment guidelines for malaria	1339	0.0 (0.0-0.3); 0.2
Stock management		
13. Availability of stock card/ledger book	1384	0.9 (0.8-1.0); 0.8
14. Correct filling of stock card	1360	0.0 (0.0-0.0); 0.0
15. Does physical count agree with stock card	1335	0.6 (0.3-0.8); 0.6
16. Stock book correctly filled	33	0.0 (0.0-0.1); 0.2
Storage management		
17. Cleanliness of the pharmacy	1380	1.0 (0.5-1.0); 0.7
18. Hygiene of the pharmacy	1384	0.4 (0.4-0.6); 0.5
19. System of storage of medicines and supplies	1384	0.3 (0.3-0.7); 0.5
20. Storage conditions	1380	0.7 (0.6-0.8); 0.7
21. Storage practices of medicines in pharmacy (stores and dispensary)	1384	0.3 (0.2-0.5); 0.4
Ordering and reporting quality		
22. Reorder level calculation	1250	0.0 (0.0-0.0); 0.0
23. Timeliness of order and distribution	63	1.0 (1.0-1.0); 0.8
24. Accuracy of Health Management Information System report	786	1.0 (0.8-1.0); 0.9
25. Filing	1329	0.5 (0.5-0.5); 0.5

#### Prescribing quality

Prescribing quality measured by five indicators (each with a maximum score of 1), was poor overall, with median indicator scores ranging from 0.0 to 0.4 of 5 (Table 3). Most facilities did not correctly implement the legally mandated *prescription recording system* that requires recording of dates, outpatient department or inpatient number, diagnosis, medicines prescribed, name of prescriber, and quantity prescribed and dispensed (half of the facilities scoring 0.0). The *rational prescribing* indicator is comprised of five sub-indicators, each with a maximum score of 0.2 and the maximum score for the indicator of 1. The five sub-indicators are average number of medicines prescribed per encounter (median score 0.0 of 0.2); percentage of medicines prescribed by generic name (median score 0.0 of 0.2); percentage of encounters with one or more antibiotics (score 0.0 of 0.2); percentage of encounters with one or more injections (score 0.1 of 0.2); and percentage of encounters with the diagnosis recorded (score 0.2 of 0.2) for an overall median score of 0.4 of 1.0*. Adherence to standard treatment guidelines* was poor for all three common conditions, with about three-quarters of the facilities scoring 0.0 on these indicators. Lowest adherence scores were found for cough and cold and diarrhea, which were often treated with antibiotics. Malaria guidelines require testing followed by treatment if needed with artemether and lumefantrine combination (first-line) or quinine (severe), but no antibiotics, and adherence was poor.

Overall median prescribing quality scores differed significantly across regions, years of baseline assessment, and estimated levels of district capacity (Table 2). Median prescribing quality scores did not differ by facility types or levels of care.

#### Stock management

Stock management is measured based on four indicators (each with a maximum score of 1) using a tracer list of 15 EMHS. Baseline results showed that about 25 % of the facilities had a score of 1.0, implying that stock cards were available for all tracer items, but not filled in correctly (median score = 0.0) (Table 3). When available and filled in, the median score on stock recording was 0.6 on indicator 15, which assesses whether the quantity of stock recorded on the stock card is in agreement with the quantity counted on the shelf. The stock book had only been introduced at the beginning of 2013. About a quarter of the facilities had a score of 0.1 out of 1 on indicator 16, which assesses correct filling in of the stock book. Median stock management scores differed significantly across regions but not across facility types, levels of care, baseline assessment years, or estimated levels of district capacity.

#### Storage management

In the area of storage management, measured using five indicators (each with a maximum score of 1), median scores ranged between 0.3 and 1.0 (Table 3). Facilities had the highest possible median score (1.0) in *cleanliness in the pharmacy* (dispensary and main store); the median score of appropriate *hygiene of the pharmacy* (with sub-indicators assessing availability of clean and functioning toilets with toilet paper and hand-washing facilities with soap) was 0.4. Three-quarters of the facilities scored 0.3 or higher in appropriate *system of storage of medicines and supplies,* where supplies are stored systematically on labeled shelves or in cupboards with stock cards. The median score for meeting standards for *storage conditions* measured with 12 sub-indicators including those assessing pest infestation, protection from sunlight, temperature regulation and monitoring, condition of the roof, adequate storage space, lockable storage, fire safety equipment, and cold storage was 0.7. Half of the facilities had a score of 0.3 or higher on the final indicator 21, s*torage practices of medicines in pharmacy,* which comprises sub-indicators that assess whether boxes are on the floor, older medicines are shelved to be dispensed first, there is a separate space for and record of expired medicines, opened tins in the dispensary have the lids on, and bottles are dated when opened. Median storage management scores differed significantly across regions, facility types, levels of care, and estimated levels of capacity, but not years of baseline assessment (Table 2).

#### Ordering and reporting

Ordering and reporting quality was measured using four indicators (each with a maximum score of 1) (Table 3). On the *reorder level calculation* indicator, which includes knowledge of the vital, essential, and necessary product classification, the median score was 0.0, while 75 % of the facilities had a score of 1.0 on the indicator assessing *timeliness of orders and distribution* which measures higher level facilities’ ordering against official schedules and the overall lead time from ordering to receipt of goods. Half of the facilities scored 1.0 or less on their *accuracy of the health management information system report*, which compares reported stock-out days to stock card information for a sample of six items. On the composite indicator of legally required *filing* systems, which includes sub-indicators measuring the use of discrepancy reports, delivery notes, previous order records, and prescription and dispensing logs, the median score was 0.5. Table 2 shows that median ordering and reporting scores did not differ across the regions and levels of care. Scores did differ significantly across facility types, years of baseline assessment, and estimated levels of district capacity.

## Discussion

Our study describes SPARS as an innovative, multipronged strategy to improve MM in Uganda and reports on the baseline facility performance in MM as measured by the indicator-based, multidomain SPARS assessment tool. With *a* median overall score of 10.3 out of 25*,* we show that assessing and building national capacity in MM are much needed in both PNFP and government facilities at all levels of care. The poor overall performance of Ugandan facilities is confirmed by results from the National Drug Authority’s good pharmacy practices inspection program, with equally low passing rates of 58 % and 57 % in PNFP and government facilities, respectively [26]. On the positive side, we note that a few facilities achieved perfect domain and total scores on the SPARS assessment tool, prior to the SPARS intervention.

### Specific MM performance in Uganda

We assessed MM performance in five domains covering appropriate medicines use (prescribing and dispensing quality) and the practices needed to ensure availability and maintain quality of EMHS. Assessment indicators have face validity, have been used globally [28–30], and were easily adapted to Uganda’s context. The MMS were trained in the purpose and use of the tool and indicators. They found the assessment tool understandable and were largely able collect data in one visit.

The assessment did not include pharmaceutical financial management indicators because HC2 and HC3 facilities receive medicines in kits free of charge. Financial management skills of staff at higher level facilities were assessed separately with different indicators (data not reported).

#### Dispensing quality

We noticed differences in dispensing quality by facility ownership. Several sub-indicators of dispensing quality are related to infrastructure and equipment, such as the availability of counting trays, drinking water, and chairs for waiting, which may explain higher dispensing quality scores of PNFP facilities that are better equipped than government facilities.

The labeling indicator had the lowest score in the dispensing domain. The indicator assesses whether the medicine is labeled with the critical information such as the name of the patient and the medicine. Several factors may explain this finding: Dispensing envelopes are sometimes out of stock, patient numbers may be too high for staff to find time to label envelopes, or staff may be using dispensing envelopes with pre-printed pictograms (without a need to fill all of the information). The latter instance resulted in a poor labeling score, but a better patient care score because a pictogram printed on the dispensing envelope effectively informs the patient when to take the medicine and how much to take. Using the same indicators, a study in Botswana found similar patient care quality, but scored much better on labeling quality, which was found to be related to the training and qualifications of the dispenser. Dispensing time in Botswana was also found to be higher—well over 100 s—and dependent on the level of care and other facility differences [31]. It was encouraging to see that in almost all cases, full courses of prescribed medicines were dispensed. That is, little rationing took place, compared to international data that highlight rationing as a problem behavior, especially when patients pay for their medicines, such as in the PNFP sector [28].

#### Prescribing quality

Prescribing performance scores were low for all levels of care. Patient demand and health workers’ inability to diagnose correctly result in symptom treatment and polypharmacy [7]. Polypharmacy, low use of generic names, and overuse of antibiotics have been found globally, and little progress has been made over time [13, 32–34]. Because prescribing habits are multifactorial, they are more difficult to change than filling out a stock card, for example. As a result, improving prescribing will require a combination of interventions [12, 33].

Adherence to standard treatment guidelines and overuse of antimicrobials are also well-known global problems, and Uganda is no exception [5, 32, 34, 35]. We did not find differences in prescribing quality based on facility ownership, while other studies have found higher use of antibiotics and lower adherence to standard treatment guidelines in the private for-profit and not-for-profit sectors [33].

#### Stock management

Correctly completed stock cards are fundamental to quantifying the medicines needed and ensuring availability. Similar to findings in Zimbabwe, where only about half of available stock cards and 13 % of stock books were filled out correctly [16], our study indicates that stock cards were available in facilities, but not filled out correctly. In the few facilities that had the newly introduced stock book, staff found it difficult to complete it correctly. Both studies confirm that keeping stock records correctly is difficult, especially when a new tool is introduced.

Stock management differed by level of care. HC4 facilities and hospitals order their EMHS every 2 months and benefit from having a well-implemented stock management system, which is not the case for HC2 and HC3 facilities that receive a bimonthly kit, making stock tracking less relevant to their day-to-day tasks. We hope through the SPARS strategy to build sufficient capacity at HC 2 and HC3 in quantification and stock management to eventually facilitate a shift from the present kit system to an order-based pull system, to optimize use of limited resources.

#### Storage management

Most storage management indicators assess facilities’ construction or equipment and scores vary by facility ownership and level of care. PNFP facilities are constructed and equipped by donors and often have more space, shelves, refrigerators, running water, and electricity than government facilities [9]. Similarly, higher level facilities are more likely to have rooms dedicated for storage and shelving.

#### Ordering and reporting

Although only higher level facilities place bimonthly orders and HC2 and HC3 receive a pre-packed kit, all health facilities are expected to submit monthly stock status reports to the national health management information system (HMIS), which the Ministry of Health has emphasized strongly, particularly in the government sector. As would be expected, performance was highest on the accuracy of data for six tracer medicines entered into the HMIS, and HC4 facilities and hospitals scored highest on the ability to calculate reorder levels, which is a routine task for them. Government facilities also scored higher than PNFP facilities, which may be explained by NMS’s 2010 introduction of strict order and delivery schedules for government facilities. During the time of the study, PNFP facilities prepared orders as needed and were not restricted to an order and delivery schedule by the Joint Medical Store (their supplier); in addition, PNFP facilities infrequently submitted reports to the HMIS in a timely fashion. We will be implementing order and delivery schedules, with door-to-door delivery and the establishment of an order budget line at Joint Medical Store for all PNFP facilities.

#### Performance variations

We found significant regional variations in the overall SPARS scores and in the scores for each domain except the ordering and reporting domains. Generally, facilities in the Northern region scored high except for stock management; whereas, those in the Eastern region had the weakest performance. Although, as mentioned below, scores across regions need to be compared with caution, we believe that civil unrest in the Northern region had previously deprived the populations there of most health service interventions; now facilities in the area are keen to catch up and make full use of the opportunities offered. The reasons for the weak performance in the Eastern region are unclear.

Scores differed by level of care. Hospitals outperformed lower level facilities both overall and in all SPARS domain areas apart from prescribing quality. Aside from being better equipped, having better structures and storage facilities for storing medicines and supplies, hospitals and other higher level facilities have dedicated staff to manage stock and storage, while fewer staff members at lower levels of care perform all MM tasks.

Baseline assessments started at the end of 2010 and peaked in 2011. Because districts and facilities were added, some baseline visits happened in 2013. Overall, MM performance at baseline differed across the years, likely due to performance differences in two domains, ordering and reporting and prescribing. From 2010 onward, the NMS continuously improved the order and delivery schedule by distributing EMHS directly to facilities. In addition, NMS reintroduced the kit supply system for HC2 and HC3 facilities. In 2011, NMS focused on increasing awareness of and adherence to the new order modes and cycles, which by 2013 had become a well-established routine. The 2012 revision of the national standard treatment guidelines, which were made available and implemented at all government and PNFP health facilities, may have contributed to improving baseline prescribing domain scores from 2010/11 to 2013. We found no correlation between scores at the baseline visit and experience of the MMS.

#### The SPARS intervention

Several studies have documented the need for a complex systematic approach to improving medicines management [2, 3, 36, 37]. When developing SPARS, we chose a multidimensional performance assessment to allow identification of diverse issues that influence MM and then to intervene with multipronged approaches to change MM behaviors and practices effectively and sustainably [14]. SPARS interventions have been implemented in Ugandan facilities since the baseline assessment. Their effects on different aspects of MM over time will be reported in a separate paper.

#### Potential study limitations

The study has the following potential limitations. Although the study facilities represent one-third of the government and PNFP facilities of Uganda, government facilities were slightly overrepresented (85 % of the sample) compared to the actual proportion of 70 % [23]. However, the PNFP sample was sufficient to detect differences of at least 10 % in baseline performance by facility ownership with 70 % power. The facilities were not randomly selected but MMS chose the facilities to be visited initially and MMS purposefully might have given priority to government facilities, well-performing facilities, or nearby facilities; any potential bias introduced by purposeful sampling by the MMS is expected to be limited because over 80 % of the facilities in the selected districts have been included in the baseline assessment. In addition, the sample’s regional representation corresponds to the SPARS rollout that started in the Western region, followed by Eastern and Central regions, and ended with the Northern region. The sample represents the distribution of facilities by level of care, with the highest proportion of facilities being HC2 followed by HC3, with fewer higher-level facilities.

Another limitation is that the baseline data are not compared to findings derived from a validated comprehensive performance assessment tool. The performance assessment tool is primarily a management not a research tool. As such, its purpose is to highlight MM domains within a facility over time so that the responsible MMS and the facility staff can focus activities for improvement. For this reason, priority was given to consistent expression and intuitive graphical representation of domain scores, rather than consistent weighting of items contributing to scores. Because domain scores are generated from different numbers of indicators, individual indicators contribute differently to the overall domain scores. In addition, some indicators could not be assessed at all facilities. In these cases, rather than penalizing facilities with a “0” score, we calculated domain scores on non-missing indicators, effectively weighing indicators differently in facilities where all indicators were assessed and those that were missing indicator scores. The majority of facilities had at least one indicator without a value. The *stock book correctly filled* indicator had the most missing values because stock books were introduced late in the baseline assessment period; therefore, only 36 facilities could be assessed on that indicator. We have not yet evaluated reproducibility or inter-rater reliability of the tool and the indicators.

In addition, baseline data was collected over a period of 4 years. We found that the facilities assessed in 2013 had a higher baseline score compared to those assessed in 2010/11. Changes in MMS experience, facility behavior, and system context over time could explain the difference. During the baseline data collection, some MMS left, new MMS joined, and overall MMS experience may have increased. Increased attention by the MMS in the district could have changed facility behavior and improved baseline scores over time (Hawthorne effect). Lastly, new medicines order processes and cycles could have effected change.

Despite these caveats, we believe that the results of our analysis of data collected with the SPARS management tool illustrate the shortcomings of MM in Uganda. The SPARS assessment tool will likely be revised over time as some indicators may become obsolete, changes may be needed to strengthen new aspects of medicines management, or when there is a need to clarify indicators. Revisions will take place after impact of the SPARS strategy over time has been evaluated.

## Conclusions

Medicines management was poor in this sample of more than 1000 public sector health care facilities in Uganda. Our baseline results highlight the need to build national capacity for monitoring and improving medicines management in both government and PNFP facilities at all levels of care. The baseline assessment of MM performance with the indicator-based multidimensional SPARS assessment tool has been implemented successfully as a first step of a long-term national process to continually measure and improve MM. Since the baseline assessment, all aspects of SPARS have been implemented. However, the effects of SPARS still have to be evaluated.

## Additional files

Additional file 1: SPARS standardized performance assessment tool. SPARS standardized performance assessment tool. Health facility supervisor’s monitoring and reporting tool for stock management, pharmacy practices and rational drug use. (PDF 937 kb) Additional file 2: SPARS medicines management performance assessment indicators. SPARS medicines management performance assessment indicators (25) by domain (5). Table of all the 25 indicators within the 5 domains. (PDF 270 kb)

## Footnotes

1 GCFLearnFree.org [Internet]. [cited 2015 Dec 8]. Available from: http://www.gcflearnfree.org/
