# Supplementary material for: Article 1: Supervision, Performance Assessment, and Recognition Strategy (SPARS) - a multipronged intervention strategy for strengthening medicines management in Uganda: method presentation and facility performance at baseline
Source: J Pharm Policy Pract. 2016 May 31;9:21. doi: 10.1186/s40545-016-0070-x (PMC4888637; doi:10.1186/s40545-016-0070-x)
Supplement: Additional file 2: — SPARS medicines management performance assessment indicators. SPARS medicines management performance assessment indicators (25) by domain (5). Table of all the 25 indicators within the 5 domains. (PDF 270 kb) [file 40545_2016_70_MOESM2_ESM.pdf]

Dispensing quality	Description
1. Dispensing time	Measures active/interactive dispensing time for 6 patients. Excludes any interruptions and time spent on communication unrelated to the patient condition or medication.
2. Packaging material	Measures availability of appropriate dispensing materials like envelopes for solid dosage forms and bottles for liquid dosage forms. Paper cones and reused bottles were considered inappropriate.
3. Dispensing equipment	Measures availability of dispensing equipment for both liquid and solid dosage forms like spoon, spatula, measuring cylinder, tablet counting tray to ensure that tablets are not counted by bare hands.
4. Services available at the dispensing area	Measures access to privacy, chairs and benches, hand washing facilities at the facility and drinking water for patients within the dispensing area.
5. Patient care	Measures discrepancy between dispensed and prescribed medications and adequacy of information provided to patients at dispensing (dose, frequency, duration, why to take and other information required for adherence to medication).
6. Labeling	Measures adequacy of information on the label (medicines name, strength, quantity, date, dose, name of patient and facility)
7. Rationing of antibiotics	Measures the practice of rationing antibiotics when in short supply. Antibiotic quantities prescribed and dispensed for 5 patients are compared to establish rationing, using amoxicillin and cotrimoxazole as examples.
**Prescribing quality**	
8. Correct use of prescription recording system	Measures appropriate recording of 10 prescriptions dispensed (date, OPD/IP number, diagnosis, medicine name, prescribers' name, quantity of medicine prescribed and dispensed).
9. Rational prescribing	This standard WHO indicator measures appropriate prescribing medicines in 20 prescriptions, assessing average number of medicines prescribed per patient, percent of products prescribed as generics, percent of prescriptions containing antibiotics, percent of prescriptions containing injections, and percent of prescriptions with diagnosis recorded.
10. Adherence to STG for diarrhea	Measures adherence to STG for non-bloody diarrhea treatment. Appropriate treatment is ORS and zinc only.
11. Adherence to STG for common cough/cold (simple respiratory tract infection)	Measures adherence to STG for cough/ cold. Appropriate treatment is optional antipyretic/analgesic without use of antibiotics.
12. Adherence to STG for malaria	Measures adherence to STG for treatment of non-complicated malaria. Appropriate treatment with antimalarials only should always follow a positive test.
**Stock management**	
13. Availability of stock card/ledger book	Measures availability of stock cards/ledger books based on basket of 15 stock items.
14. Correct filling of stock card	Measures correct filling of stock cards/ledger books (medicines name, strength, dosage form, average monthly consumption, special storage conditions).
15. Does physical count agree with recorded stock card balance.	Measures whether stock balance according to stock card agrees with counted physical stock.
16. Stock book correctly used	Measures correct use of stock book (all column information is appropriately filled and calculated, including average monthly consumption and quantity to order).
**Storage management**	
17. Cleanliness of the pharmacy	Measures cleanliness of the dispensary and main store (floor, wall, shelves and medicines are checked).
18. Hygiene of the pharmacy	Measures availability, functionality, and hygiene of designated sanitary facilities for dispensary staff (toilet, toilet paper, hand washing and soap).
19. System for storage of medicines and supplies	Measures if medicines in the facility are stored on shelves/cupboards in an appropriate and systematic manner and the shelves are labeled.
20. Storage conditions (main store)	Measures appropriate physical storage conditions and steps taken to assure quality and safety of medicines in storage (sign of pest, protection from light, temperature monitoring and regulation, roof condition, storage space, lockable storage, fire safety equipment, cold storage, separate storing of medicines/vaccines appropriately in refrigerator, recording temperature in refrigerator).
21. Storage practices of medicines in pharmacy (stores & dispensary)	Measures adherence to good storage practices (incorrect storage on the floor, expired items recorded and stored separately, FEFO, opened bottles labeled with opening date, and lids on all containers).
**Ordering and reporting**	
22. Reorder level calculation	Measures ability of the facility to correctly calculate reorder quantity.
23. Timelines of order & distribution	Measures adherence to order and delivery schedules (only applicable for higher level facilities).
24. Accuracy of HMIS reports	Measures if the health facility staff update the HMIS 105 report with accurate information on medicines availability during the previous month from stock management records. Stock card and HMIS 105 information are compared for consistency for a basket of 6 EMHS.
25. Filing	Measures appropriate filing of previous orders, delivery notes and discrepancy reports

Notes: OPD=outpatient department; IP=inpatient; ORS=oral rehydration solution; STG=standard treatment guidelines; FEFO=First expiry first out; HMIS=Health Management Information System; EMHS=essential medicines and health supplies;
